# Adjunctive Approaches to Aphasia Rehabilitation: A Review on Efficacy and Safety

**DOI:** 10.3390/brainsci11010041

**Published:** 2021-01-02

**Authors:** Chiara Picano, Agnese Quadrini, Francesca Pisano, Paola Marangolo

**Affiliations:** 1 Department of Psychology, University La Sapienza, 00185 Rome, Italy; chiarapicano1@gmail.com; 2 Aphasia Laboratory, IRCCS Santa Lucia Foundation, 00179 Rome, Italy; agnese.quadrini@gmail.com; 3 Department of Humanities Studies, University Federico II, 80133 Naples, Italy; francescapisano00@virgilio.it

**Keywords:** post-stroke aphasia, aphasia rehabilitation, pharmacological approach, virtual reality, transcranial direct current stimulation (tDCS)

## Abstract

Aphasia is one of the most socially disabling post-stroke deficits. Although traditional therapies have been shown to induce adequate clinical improvement, aphasic symptoms often persist. Therefore, unconventional rehabilitation techniques which act as a substitute or as an adjunct to traditional approaches are urgently needed. The present review provides an overview of the efficacy and safety of the principal approaches which have been proposed over the last twenty years. First, we examined the effectiveness of the pharmacological approach, principally used as an adjunct to language therapy, reporting the mechanism of action of each single drug for the recovery of aphasia. Results are conflicting but promising. Secondly, we discussed the application of Virtual Reality (VR) which has been proven to be useful since it potentiates the ecological validity of the language therapy by using virtual contexts which simulate real-life everyday contexts. Finally, we focused on the use of Transcranial Direct Current Stimulation (tDCS), both discussing its applications at the cortical level and highlighting a new perspective, which considers the possibility to extend the use of tDCS over the motor regions. Although the review reveals an extraordinary variability among the different studies, substantial agreement has been reached on some general principles, such as the necessity to consider tDCS only as an adjunct to traditional language therapy.

## 1. Introduction

Aphasia is an acquired language disorder which occurs in about one-third of people suffering from left-cerebral artery stroke [1,2]. It impairs the person’s ability to process language with heterogeneous symptoms varying in terms of severity and degree of involvement across the different linguistic modalities, including the expression and comprehension of language, reading and writing [3]. Variation in the severity of expressive impairments, for example, may range from the patient’s occasional inability to find the correct word to telegraphic and very reduced speech output [4]. Aphasia is one of the most serious socially disabling consequences after stroke. Indeed, the language impairment determines loss of autonomy and of independence, thus dramatically compromising social relationships [5].

After an initial spontaneous recovery, most notably during the first 2–3 months following stroke onset, language improvement can occur in response to behavioral training also in the chronic phase [6].

The recent progress in neurorehabilitation, which takes into account the mechanisms underlying cerebral reorganization [7,8,9], has suggested that a crucial predictor for positive language outcomes is to provide an intensive language training which lasts several hours per week (5–10) [10].

Unfortunately, most of the persons with aphasia (PWA) cannot receive the recommended amount of training estimated [10,11]. Indeed, the healthy system has limited economic resources which cannot guarantee for language intensity and not all patients can afford the costs to have access to the private health care services. As a consequence, speech-language therapies do not always result efficacious and many patients are left with some degree of language deficits [12,13].

Therefore, other rehabilitation methods which act as a substitute or as an adjunct to traditional approaches are urgently needed in order to maximize the language recovery process. In this context, unconventional approaches which increase treatment effectiveness, by either improving the total amount of learning achieved (e.g., number of correct responses or by speeding up the learning process (e.g., vocal reaction times), have been proposed [14]. In particular, to date, three approaches appear to be promising for positive language outcomes: the pharmacological approach, Virtual Reality (VR), and Transcranial direct current stimulation (tDCS).

With regard to the first approach, despite the recently waned interest for pharmacotherapy in aphasia, it has been demonstrated that some types of drugs, acting on neurotransmitter activity, promote adaptive neuroplasticity and network remodeling [15]. Indeed, they seem to rebalance the altered neurotransmitters, thus improving cognitive performance [16,17].

With regard to Virtual Reality, the hypothesis has been advanced that, the use of virtual contexts promotes the ecological validity of language therapy which seems to be a key predictor for language recovery [12].

With regard to tDCS, there is substantial agreement on its use as an adjunct to language therapy [18]. Indeed, to date, the application of tDCS has been extended in domains other than the treatment of word finding difficulties [19,20,21,22,23], such as the recovery of articulatory deficits [24,25,26] and speech production [27,28,29,30].

The purpose of the present review is to gain an understanding of the existing unconventional approaches for aphasia rehabilitation and to provide a critical overview of the state of the art.

## 2. Pharmacological Approach

In post-stroke aphasia, and all stroke cases, there is an acute stage followed by a period of spontaneous recovery in which many symptoms can regress. Immediately after the cerebrovascular event, a series of metabolic neural reorganizations occurs [31]. These metabolic changes include ischemic penumbra, a dysfunctional cerebral area surrounding the infarct due to reduced blood flow [32]; this area can be saved because its neurons could remain alive for some time. Another event is the diaschisis, which consists of neurophysiological changes (with inhibitory or excitatory effects) directly caused by a focal injury in anatomically intact areas distant from the lesion [33]. Lastly, during this period of spontaneous recovery, phenomena of brain plasticity also take place [34].

The interest in the pharmacological treatment for post-stroke aphasia arises from the hypothesis that drugs could act on those post-stroke mechanisms, thus, promoting language recovery [35]. Indeed, one of the mechanisms by which stroke causes aphasia is by interrupting major neurotransmitter pathways, such as the cholinergic fibers, which connect the brainstem and basal forebrain to perisylvian language regions [36,37].

A PubMed search on pharmacological treatment in post-stroke aphasia (keywords: Aphasia and drug therapy/drug treatment/drug effect/pharmacotherapy/neuropharmacological treatment/pharmacological treatment; aphasia treatment and piracetam/bromocriptine/amphetamine/donepezil/galantamine/memantine/levodopa/L-dopa) produced 142 results. We selected 31 articles, and excluded 2 articles without full text and 109 articles which comprised reviews, non-English articles, and articles in which aphasia was a secondary symptom of other pathologies (e.g., dementia).

In the next paragraph, we present the main drugs used as an adjunctive or substitutive treatments for aphasia. All the published studies and details on their methodological aspects are summarized in Table 1. 

### 2.1. Drugs Acting on GABA, Acetylcholine, and Glutamate Systems: Piracetam, Donepezil, Galantamine, and Memantine

Piracetam is a derivative of GABA (γ-aminobutyric acid), but its mechanism of action appears to be unrelated to the properties of this neurotransmitter.

Several well-designed trials have investigated the combined effect of piracetam with language treatment in post-stroke aphasia [39,40,41] administering a dose of 4800 mg/day in acute post-stroke patients. In all studies, significant improvements were found in the piracetam group compared to the placebo group. In particular, compared to baseline, better performance was observed in written language [40,41], repetition [41] and spontaneous speech [39].

Differently from previous studies, Güngör et al. [38] investigated the effect of piracetam as a substitutive treatment of speech therapy. Fifteen patients received oral piracetam as a daily dose of 4.8 g for 24 weeks, while fifteen patients received a placebo with the same dose. After the treatment, a significant improvement in auditory comprehension was reported which did not persist at six months after the end of the therapy.

The exact mode of action of the piracetam is still unknown but there is increasing evidence that its underlying effect is to reestablish cell membrane fluidity. As a consequence, piracetam could have a neuroprotective action, it may restore neurotransmission and enhance neuroplasticity [69]. Moreover, the hypothesis has been advanced that piracetam facilitates the transcallosal transfer of information from one hemisphere to the other, promoting relearning of degraded linguistic knowledge and learning of compensatory strategies [32].

In summary, piracetam could act as a potential drug for language recovery in aphasia but, to date, the number of studies is too small in order to reach definite conclusions on its real therapeutic efficacy.

Donepezil is a reversible acetylcholinesterase inhibitor [70]. Acetylcholinesterase is an enzyme that catalyzes the breakdown of acetylcholine, so the drug, increasing the presence of this neurotransmitter in the synaptic cleft, enhances its action [71]. Cholinergic pathways are vulnerable to vascular damage and stroke could interrupt cholinergic projections in cortical sites necessary for language processing [72].

Studies about donepezil therapy in aphasia have reported contrasting data. In three studies, Berthier et al. [43,46,47] have shown a positive effect of the drug in repetition, picture-naming, and speech production with mild adverse effects (irritability, increased sexual drive, insomnia, headaches, dizziness, muscle cramps). Dávila et al. [42] found an improvement in fluency, spontaneous speech, naming and phrase repetition but, in this case, no side effects were found. In this last study, in our opinion, the absence of side effects was probably due to the young age of the participant (9 years old) and the fact that the dosage of the drug was really small (5 mg/day).

The patient of Yoon et al. [45] improved in spontaneous speech and comprehension, while, in Woodhead et al.’s study [44], worse comprehension in the drug condition compared to placebo was, unexpectedly, found. The hypothesis advanced in this last study was that cognitive-enhancement drugs can have opposite effects on cognitive tasks, as an inverted U-shaped relationship: hence, if acetylcholine stimulation is already high in the auditory cortex, a further increase through donepezil might worsen the patient’s performance [73].

It is interesting to note that in all of these studies the patients had chronic aphasia, so donepezil does not seem to act on spontaneous recovery mechanisms which occur in the acute stage. It has been suggested that donepezil plays probably a role in language recovery enhancing the effect of speech therapy: indeed, an increase in cholinergic transmission into the cortex could boost attention and learning, facilitating the encoding of verbal stimuli and filtering of irrelevant ones [36,74].

Galantamine, like donepezil, is a reversible acetylcholinesterase inhibitor that modulates multiple subtypes of nicotinic acetylcholine receptors [48]. It is currently being researched for the treatment of Alzheimer’s disease [75] and, in some studies, it was also administered in chronic aphasia.

Hong et al. [48] conducted an open-label trial on 45 chronic aphasic patients, administering galantamine without any adjunctive therapy, and they observed a significant improvement in spontaneous speech, comprehension, and naming.

Galantamine augments NMDA-evoked currents in rat cortical neurons and potentiation of the activity of NMDA receptors is considered to be partially responsible for the improvement of cognition, learning and memory in Alzheimer’s patients [76]; however, only one trial is not enough to establish its efficacy in post-stroke aphasia.

Memantine is an uncompetitive NMDA receptor antagonist; thus, it reduces neurotransmitter glutamate activity. Two double-blind placebo-controlled studies [49,50] have investigated the effects of memantine on chronic post-stroke aphasia both considering the drug as adjunctive treatment to constraint-induced aphasia therapy (CIAT).

Barbancho et al. [49] observed a general increase in language functions. Berthier et al. [50] found a significant improvement in spontaneous speech, comprehension, naming and everyday communication. Interestingly, these beneficial effects of memantine persisted at the 6-months follow-up evaluation.

The effect of this drug is peculiar because other NMDA receptor antagonists (such as ketamine) have serious negative effects [77]. The hypothesis is that the memantine-specific mechanism of action on NMDA receptors could provide both neuroprotection and reverse deficits in learning and memory [78]. It seems that it can augment synaptic plasticity and long-term potentiation in spared networks through enhancing activity-dependent learning mechanisms in language-related brain regions [78].

Memantine has already the authorization to be used in Alzheimer’s disease because these patients manifest an over activation of glutamate receptors, in particular, the NMDA ones [78]; but further studies to confirm its therapeutic effect in post-stroke aphasia are also needed.

### 2.2. Drugs Acting on Monoaminergic Systems: Levodopa, Bromocriptine, and Amphetamines

Several studies investigated the therapeutic potential of monoamine agonists, especially dopamine, in aphasia recovery. The rationale is broad: dopamine pathways are present in many cerebral areas involved in language processing and damage into these areas could cause aphasic symptoms [71].

The second explanation concerns LTP (Long Term Potentiation) mechanisms, in particular, the role of NMDA receptors over these mechanisms. In fact, NMDA-dependent LTPs are involved in different forms of memory [71]. Nevertheless, for long-lasting effects, neither NMDA receptors alone nor combined with other glutamatergic agonists are sufficient and an adjunctive mechanism is needed to exert the intended effect [79,80]. This additional mechanism could be related to monoamines′ action; indeed, two studies have already shown that depletion of serotonin or catecholamine could modulate LTP mechanisms in the dentate gyrus in rats [81,82]. Thus, in order to maintain long-lasting therapeutic effects for aphasia recovery, an increase in monoamines’ action might be the responsible factor.

Levodopa (L-dopa) is a dopamine precursor. Dopamine modulates attentional processes, working memory, long-term potentiation, neuronal growth and it improves word learning in healthy humans [83,84,85,86,87]. Only three studies investigated the effect of this drug on aphasia.

Seniów et al. [53] administered L-dopa as adjunctive treatment to speech-language therapy (SLT) in acute aphasic patients and found an improvement in verbal fluency and repetition compared to the placebo group. Leemann et al. [52] and Breitenstein et al. [51], in contrast, found no significant differences in the L-dopa-group combined with high-intensity language training compared with the placebo-group (only high-intensity language training).

According to Breitenstein and colleagues [51], “the negative effect could be due to a behavioral “ceiling” effect induced by the high intensity training (four hours per day), touching the physiological plasticity limit”. This hypothesis is supported by prior findings on significant L-dopa effects for language recovery combined with a daily intensive treatment of forty-five minutes in subacute stroke patients with circumscribed anterior lesions [53] but not in subacute stroke patients undergoing a speech-language therapy regime of two hours per day [52].

Bromocriptine is a dopamine agonist which stimulates D2 dopamine receptors non-adenyl cyclase-linked [88]. Based on the effects of dopamine, various trials investigated the effect of this drug on post-stroke aphasia as a substitute to traditional language therapy.

Gupta and Mlcoch [60] found an improvement in mean length utterance, verbal fluency, and naming; Raymer et al. [55] reported significant improvement in verbal fluency and Gold et al. [56] in word retrieval. Sabe et al. [58], Ashtary et al. [54] and Gupta et al. [59], conversely, did not find any effects. Bragoni et al. [57] investigated the efficacy of bromocriptine alone and as an adjunct to traditional therapy and found a significant improvement in reading comprehension and verbal latency (the time delay in starting to declare words expressed in seconds) in both experimental conditions. When the drug was combined with language treatment, a significant improvement in repetition was also found. However, in this last study, the patients reported several severe adverse effects: epileptic seizures, atrial flutter, atrial fibrillation, visual hallucinations, mild nausea and a syncopal episode. In agreement with de Boissezon et al. [89], we believe that it is not possible to conclude on any effective influence of bromocriptine on aphasia symptoms and the encouraging reports seem rather anecdotal.

The term “amphetamines” generally refers to amphetamine proper and all its relatives, like MDMA (or ecstasy) methamphetamine, dextro-amphetamine [90]. The major mechanism of action of these substances is to block and/or reverse dopamine, norepinephrine, and serotonin reuptake transporters, causing an increase in these neurotransmitters in the synaptic cleft [91,92]. The result of this action is a psychoactive effect; indeed, they can increase arousal, vigilance, wakefulness, and attention [93]. For this reason, amphetamines are currently the most widely misused stimulants [94].

Like other abused drugs, amphetamines cause tolerance and psychological addiction and cognitive impairments could arise in habitual users [95,96,97]. Nevertheless, they have already been approved for the treatment of Attentional Deficit Hyperactivity Disorders (ADHD) and narcolepsy [91]. Their possible therapeutic effects are also currently being studied for many disturbances like eating disorders, multiple sclerosis, and schizophrenia [91,98], as well as motor recovery [99] and aphasia.

Walker-Batson and her research team [68] published the first single case study in an acute patient on the use of amphetamines (dextro-amphetamine) in aphasia as an adjunctive treatment to Speech-Language Therapy (contrastive stress drills coupled with traditional speaking, reading, and auditory comprehension tasks). A significant improvement in different aspects of language was found and, in particular, in verbal fluency. Some years later, the same authors published an open-label pilot study [67], but the results were not significant. After these preliminary studies, other trials reported contrasting results in chronic post-stroke aphasia. Whiting et al. [63] and Spiegel and Alexander [62] found some general but non-significant improvements, administering dextro-amphetamine adjunctively to traditional speech therapy [62] and computer-based speech therapy [63]. McNeil et al. [66] did not obtain significant effects even with the administration of selegiline (a derivative of methamphetamine).

Interestingly, very recently, Keser et al. [61] investigated the effect of dextro-amphetamine combined with tDCS and Melodic Intonation Therapy (MIT). Ten subjects with chronic nonfluent aphasia underwent two experiments where they received dextroamphetamine or placebo along with tDCS and MIT over two days, separated by a ten-days washout period. The authors observed a general significant improvement in aphasia severity when the different therapeutic approaches were combined together and no side effects, demonstrating that triple combination therapy is safe.

Several years after the first open-label reports, Walker-Batson et al. [65] published a randomized, double-blind, placebo-controlled trial and found a significant and positive effect of dextro-amphetamine in acute post-stroke aphasia. Similar results were found by Stefanatos et al. [64]. It is interesting to note that, in this last study, the drug treatment was substitutive to traditional therapy and it was particularly effective also in auditory attention. In all of these trials, no severe adverse effects were observed, only episodes of mild insomnia have been reported [61,67].

D-amphetamine seems to be more effective during the acute stage than in the chronic stage, but only a few studies have investigated the effects of this drug in patients with chronic post-stroke aphasia. D-amphetamine plays a role in synaptogenesis; thus, it seems that the treatment with d-amphetamine, combined with a language task, can selectively regulate the growth of neurites, in the brain circuits underlying the behavioral function tested, despite the loss of the original pathways [100]. However, as for the other pharmacological approaches, further clinical trials are required in order to clearly understand its effectiveness.

### 2.3. Conclusions

In this section, we examined the current state of evidence regarding the major drugs used for aphasia treatment. In most studies, the drug therapy was administered in combination with conventional language protocols; bromocriptine is the only drug that has been studied primarily as a substitute for speech therapy, but there is no evidence of positive effects and possible side effects of this drug are alarming [57,58,59].

In general, the main problem of all of these studies is the lack of a sufficient number of double-blind, placebo-controlled trials with large sample size. Another problem lies on the fact that our knowledge regarding the precise therapeutic mechanisms of action of each drug is still insufficient, together with their side effects and efficacy over a long period of use.

Thus, further studies on the above reported drugs are needed in order to reach definitive conclusions. Moreover, it would be also interesting to include some studies on the role of other drugs in post-stroke aphasia, such as Fluoxetine, Inosine, and Citicoline. Indeed, Fluoxetine enhances serotonin levels in certain brain areas; while Inosine exerts powerful axon-promoting effects; finally, Citicoline is involved in the synthesis of myelin leading to improvement in neuronal membrane integrity. Right now, the exact mechanisms of action of these drugs are not completely known but they seem promising since they have already been successfully used in post-stroke patients for motor recovery [101].

## 3. Virtual Reality Approach

Digital therapeutics is a newly emerging concept of therapeutic approach in the healthcare system which includes smartphones, personal digital assistants, computer programs, tablets, and virtual reality (VR) [102]. Indeed, the use of digital devices could facilitate high-intensity programs and help patients maintaining their improvements [102]. This is especially true for tablet or computer self-administered therapies delivering several language exercises on words and sentences listening, reading, and writing and also for speech fluency [103,104,105,106,107]. Home practice programs could also solve the problem of travelling for patients who live far away from rehabilitation centers, especially those who have also motor impairments [108].

To date, there is a growing body of studies on the use of these technologies, and, in particular, VR, for the treatment of post-stroke impairments [109], such as aphasia.

Development of VR applications for the rehabilitation of aphasia is still at its early stage ([110,111,112,113]; see for a review [102]). This involves a computer-generated simulation of 3D environments with which the user can experience a semi-immersive interaction that may encourage language practice in real context communication settings. Typically, an individual entering a virtual environment feels a part of this world, and she/he has the opportunity to interact with it almost as she/he would do in the real world. Accordingly, the latest Cochrane review on speech and language therapy following stroke concluded that therapy should enhance functional communication in ecological contexts [12]. Indeed, a common observation regarding PWA is that they can communicate much more than their linguistic abilities would suggest. Therefore, a more ecological approach aimed at restoring the patient’s ability to communicate in different daily contexts, such as VR, has been proposed [23,114,115,116].

Another important advantage of VR is that the inclusion of a virtual therapist does not require the physical presence of a clinician; thus, it allows the patient to practice at any time [117,118]. This would further reduce the costs of the therapy and, thus, ensure intensive treatment.

A PubMed search on post-stroke aphasia and VR (keywords: aphasia and VR/virtual reality) produced 24 results. We selected 8 articles, and excluded 16 articles which comprised reviews or articles in which aphasia was a secondary symptom in other pathologies (e.g., dementia).

In the next paragraph, we report a presentation of the VR studies published until now for the treatment of aphasia. All the reported studies and details on their methodological aspects are summarized in Table 2. As far as we know, there were no reported side effects in any of the studies.

Marshall et al. [113] designed a semi-immersive virtual world called EVA Park, a virtual island with houses, a cafe, a restaurant, a health center, a hair salon, a tropical bar, and a disco. Users can visit the places, interact with the several objects available, and communicate with other users with a personalized avatar. The quasi-randomized controlled design compared a group that received immediate EVA Park intervention with a waitlist control group. The content of the intervention was largely driven by participants that set at least three context-bound goals such as ordering food in a restaurant or making a doctor’s appointment. Outcomes showed significant improvement in functional communication, but no significant effects in verbal fluency, word-finding in conversation, narrative, and communication confidence.

Marshall et al. [122] reported another EVA-Park-based intervention in two single cases. Treatment consisted of noun retrieval therapy for the first patient and Verb Network Strengthening Treatment for the second one, conducted remotely in EVA Park delivered over five weeks. Both patients showed excellent compliance and positive views about EVA-Park-experience and they had a significant increase in naming after the end of therapy which in one case was not maintained at five-weeks follow-up.

Very recently, Marshall et al. [120] published a randomized controlled design in which a group therapy was virtually implemented in EVA Park, with patients, volunteers, and coordinators present in the VR environments. Two intervention groups were randomized to an immediate therapy condition and two were randomized to a delayed condition. The comparison of scores was made when the immediate condition had received the intervention, and the delayed group had not yet undergone the treatment. The study aimed to investigate whether it is feasible to deliver group social support to people with aphasia via a multi-user, VR platform. It also explored the indicative effects of intervention and the costs. Possible improvements in wellbeing and communicative success were also measured. No significant changes were found on any of the outcome measures, although the study was not powered to detect these. The results suggest the need to implement the study with a larger trail of remote group support, using VR.

A pilot study explored the remote delivery of storytelling intervention in EVA Park on three patients with chronic aphasia [123]. Participants practiced with a therapist to generate a story based on video stimuli and then, met a volunteer who was unfamiliar with the story to tell it in their own words. Following the intervention, two participants showed an increase in the number of content words produced in storytelling.

Grechuta and collaborators published a pilot study prior [124] to a randomized controlled trial [110]. In both studies, they investigated a new VR intervention with motion tracking. Patients interacted with the system by performing horizontal arm movements over a table surface. These movements were tracked in real-time and mapped onto avatars′ upper limbs, allowing the interaction with virtual objects. This method was based on the assumption that motor planning and control circuits seem to be also involved in the comprehension and perception of language; in particular, during speech perception, specific motor circuits are recruited that reflect phonetic features of the speech sounds encountered [125]. The paradigm involves two patients interacting with each other by requesting objects, placing their hand on top of them, and verbally requests them. In the pilot study [124], a silent video representing the correct pronunciation of the target word aided the verbal request and the results showed that these silent visuomotor cues facilitated word retrieval and verbal execution. In the second study [110], the authors compared the effect of this training with Intensive Language-Action Therapy (ILAT) and they found that both groups improved in speech production, auditory comprehension, communicative effectiveness in everyday life, and lexical access, but only the experimental group maintained the improvements at sixteen-weeks-follow-up.

Maresca et al. [121] investigated the effect of a VR-based intervention using tablets compared to traditional therapy (the same exercises delivered by tablet but using paper-pencil tools). The training includes exercises in which the patient interacts with objects and virtual scenarios through a touch screen. The exercises concerned tasks of naming, composition, writing, and rewriting suggested by acoustic, textual, or visual items. Results showed that the experimental group improved in all investigated tasks except in writing, while the control group improved only in comprehension, depression, and quality of life.

Very recently, Giachero et al. [119] explored the effectiveness of conversational therapy paired with semi-immersive VR-experience compared to therapy without VR support in two different groups of chronic aphasia patients. The effectiveness of the treatment was evaluated not only on language skills but also through scales for measuring different psychosocial aspects. Participants were asked to explore different virtual everyday scenarios which included cognitive exercises with language, memory, and attentional tasks. The interaction among patients was mediated by a speech therapist who operated in the VR scenario through the use of a personal computer. After the treatment, no significant differences in the different measures were present between the two groups. However, the amount of improvement in the different areas was distributed over far more cognitive and psychological aspects in the VR group than in the control group. Indeed, the within-group comparisons showed a significant enhancement in different language tasks (i.e., oral comprehension, repetition, and written language) only in the VR group. Interestingly, after the treatment, significant gains were also found, in the VR group in different psychological dimensions such as in their self-esteem and emotional and mood state.

### Conclusions

From the studies reported above, it seems likely that the use of VR, although it is still in its infancy, is promising. First, in the case of self-administration treatments delivered at home, this would engage the patient for several hours a day; thus partly resolving loneliness and social isolation often experienced by PWA. Indeed, the possibility for the patients to practice on their own and to take care of themselves would enhance their responsibility, their self-esteem, self-efficacy, and independence. This would also guarantee the possibility to undergo an intensive treatment program, which is one of the key predictors in aphasia rehabilitation in order to obtain the best language outcome.

Secondly, interventions would occur in virtual everyday contexts, thus enhancing the ecological validity of the treatment protocols [12,119]. Finally, the impact of VR for language recovery, as already pointed out [110,124], is in line with a recent perspective which considers the language faculty as represented in a multimodal dimension where word semantics contain sensorimotor properties, which rely on areas not previously hypothesized by the traditional approach, such as the sensorimotor network [119,126]. Within this view, these sensorimotor properties are more easily enhanced in a VR environment than in a usual language therapy setting.

## 4. Transcranial Direct Current Stimulation (tDCS) Approach

Transcranial Direct Current Stimulation (tDCS) is a non-invasive technique which, in these last years, has been considered as a new potential adjunctive therapy for neurological disorders [127,128]. Indeed, tDCS induces a weak electrical current (1–2 mA) through a pair of surface electrodes applied over the scalp, which modulates cortical excitability over a period of time (usually 20 to 30 min), thus potentiating the effects of the treatment [129,130]. It has been shown that the electrical current employed in tDCS acts on the resting membrane potentials increasing or decreasing the probability that neural firings are triggered; anodal stimulation induces depolarization of the neuronal membrane, thus, increasing the spontaneous neuronal firing rate, whereas cathodal stimulation leads to neuronal hyperpolarization and inhibition [130,131]. These effects may last for minutes to hours depending on the intensity, polarity, and duration of stimulation [130]. In all the experimental protocols, two different stimulation conditions are usually employed: a real condition and a placebo condition. During the real condition, the anode or cathode are applied over the target area to explore respective excitatory or inhibitory effects. Conversely, during the placebo condition, called sham condition, the stimulator is turned-off after few seconds. The comparison of these two stimulation conditions is necessary in order to ensure that the patient′s behavioral changes are specifically attributable to stimulation [18]. A growing body of evidence has already suggested that tDCS provides a supplementary treatment approach for language deficits in patients with chronic stroke-induced aphasia [4,18].

A PubMed search on “tDCS and aphasia” (keywords: tDCS and aphasia) produced 196 results. We selected 37 randomized-controlled trials in post-stroke chronic aphasia and excluded: 9 studies on acute patients, 8 single-case studies, 26 studies on primary progressive aphasia, 16 studies on healthy subjects, 65 reviews, and 35 articles on other pathologies and stimulation approaches (e.g., dementia, transcranial magnetic stimulation (TMS)).

From these selected studies, an extreme variability emerged with respect to electrodes location, current density, number of tDCS sessions, length of follow-ups and the language protocols and outcome measures applied. Thus, this methodological heterogeneity makes it difficult to establish universal principles on the use of tDCS in aphasia. However, we will report some general assumptions on which there is substantial agreement in the literature.

In the next paragraph, a brief overview of the tDCS studies published until now for the treatment of aphasia is reported. All the reported studies and details on their methodological aspects are summarized in Table 3.

Similar to stroke motor function parameters, in tDCS studies, the electrode placement aims to restore the interhemispheric unbalance between the residual left language areas and the right hemispheric ones [157,158]. Thus, two broad approaches have been usually adopted: facilitation of activity in lesioned or perilesional areas through anodal stimulation [19,20,21,22,23,24,27,28,30,134,135,136,138,139,141,142,143,144,145,146,149,150,151,153,156] or inhibition of the intact right hemisphere to diminish abnormal transcallosal inhibition through cathodal stimulation [20,133,137,140,145,152,154]. There were no reported side effects in any of the studies, even when tDCS was applied in a multiple session paradigm [127,128]. Together with these two approaches, more recently, dual stimulation has been proposed in which the left and right hemisphere are simultaneously targeted with anodal and cathodal stimulation, respectively, to enhance activity into the left perilesional cortex [25,26,29,132]. Indeed, a very recent modeling study by Galletta et al. [159], which compared the most used electrode montages in tDCS aphasia studies, has concluded that dual stimulation exerts the highest electric field magnitude over the left perilesional areas. Thus, if our aim is to boost the recovery process into the left perilesional areas because, according to the literature, we believe that language recovery takes anyway place into the left hemisphere [160] through dual stimulation we can obtain this effect.

Studies also included a mix of different types of aphasia classification (reporting either the patients’ scores or classifying their symptoms and/or considering their speech fluency) contributing to their extreme heterogeneity [127]. The classical modular approach has been most often adopted; thus, positioning the active electrode either over the left Broca’s area [19,21,23,24,25,26,27,28,29,30,132,135,142,144,145,150,151,156], the left Wernicke’s area [19,21,22,23,28,30,138,139,152,153] or the right homologues [20,133,137,145,147,152,154,155]. Indeed, most of the research has proven that anodal stimulation over these areas combined with language therapy increases different aspects of language. Indeed, the Broca’s area is a crucial part of the language network involved in different aspect of language processing [161,162,163] and it also plays an important role in the recovery of units with high communicative value (i.e., content units) [28,30].

More specifically, a significant improvement has been shown in noun naming after anodal tDCS over the left frontal gyrus [27,29,145,150,151,156] the left temporal gyrus [21,22,23,138,139,153] or the right homologues [137,145,152,154]. Some other studies have targeted the left inferior frontal gyrus in order to improve verb naming [21,23,27,28,29,148], speech fluency [27,28,29,30], repetition [24,25,26,135] and reading [142]. A couple of reports [147,155] have also investigated whether melodic intonation therapy (MIT) combined with anodal stimulation over the right inferior frontal gyrus would increase articulatory difficulties in post-stroke aphasia. Both studies showed positive results with a greater improvement in syllables, words, and sentences repetition after the active condition [147,155].

More recent studies, overcoming the classical approach which considers the language faculty mostly represented into the Broca and Wernicke’s area, have investigated the impact of tDCS over different brain regions, and, in particular, over the motor regions [134,136,143,146]. In the study by Meinzer et al. [146], anodal tDCS was delivered twice daily for two weeks over the left motor cortex combined with a computer-assisted naming therapy. After the treatment, an increase in naming accuracy was found both for trained and untrained items which persisted at six months follow-up [146]. Another study has targeted the cerebellum resulting in an improvement in verb generation after cathodal DCS [140].

Finally, two studies have targeted the dorsolateral prefrontal cortex [20,141] in order to investigate the role of executive functions on lexical access. Pestalozzi et al. [141] combined left dorsolateral prefrontal DCS with picture naming, verbal fluency, and word repetition tasks for two sessions. After the treatment, improvements in phonemic verbal fluency and picture naming were found, but only for very high-frequency words.

As reported in Table 3, the studies were not homogeneous on the number of sessions included varying from one [136,141,143,144,151] to five [21,22,23,24,25,132,133,135,137,140,142,153,154,156] to fifteen sessions [26,138,139,147]. While for current intensity, there was a substantial agreement to use 1 mA [19,21,22,23,24,25,28,30,133,134,135,138,139,141,143,146,148,149,152,153,156] to 2 mA [20,26,27,29,132,133,136,137,140,142,144,145,147,151,154]. Unfortunately, almost half of the reported studies did not include follow-up sessions [19,27,30,132,136,141,143,144,148,151,154,155] and, in the remaining studies, tDCS effects were measured up to 1–4 weeks after the treatment [20,21,22,23,25,26,28,29,133,135,137,139,140,145,147,149,150,152,153,156] but see [20,24,134,138,142,145,146]. Anyway, beyond this variability, the literature agrees on some aspects which might assure the long-term maintenance of stimulation efficacy. Long term effects are more easily obtained stimulating the subjects for several consecutive days [20,21,22,23,24,25,26,28,29,133,134,135,137,138,139,140,142,145,146,147,149,150,153,156]. Indeed, the hypothesis underlying multiple session paradigms is that short-lasting effects from a single session will accumulate with repeated sessions and eventually lead to a permanent improvement in the treated function [18] and/or on untrained materials [146]. It has also been suggested that higher current intensity (i.e., 2 mA) brings greater benefits than lower (i.e., 1 mA) [133]. Fiori et al. [133] highlighted that the systematic determination of stimulation intensity appears to be crucial for obtaining relevant effects. The authors found a significant improvement in verb naming only after cathodal high definition (HD)-tDCS at 2 mA compared to 1 mA.

One important point on which there is a total agreement is that tDCS must be delivered with concomitant language treatment [18,127]. Indeed, the rationale of the treatment is to potentiate the training [18].

Since anomia is the most common symptom in aphasia, most crossover studies focused on word recovery by combining tDCS with naming task, from noun treatment [20,21,22,23,138,143,144,145,146,149,150,151,152,153,154,156] to verb treatment [21,23,133,140,148,150]. tDCS has also been combined with repetition task [24,25,26,135], Melodic Intonation Therapy [147,155], and conversational therapy in order to enhance speech fluency [27,28,29,30,132,134].

In summary, although the results of these studies look very promising, it is worth noting that most of the studies have used a treatment approach (i.e., computerized matching, picture naming [20,21,22,23,139,141,143,144,145,150,151,152,154] which has not always been considered effective in the literature [12]. However, these approaches have been combined with tDCS because they offer a highly constrained replicable treatment method, possibly aimed at promoting repetition and intensity of the treatment, which are both aspects known to promote neuroplasticity [164]. Only very few studies have considered combining tDCS with evidence-based treatment [25,29,30,147,148,155]. Indeed, it is possible that pairing noninvasive brain stimulation with appropriate cognitive tasks and behavioral therapies may increase the “behavioral resolution” of the stimulation procedures. A final missed point was the lack of outcome measures for quantifying the improvement in functional communication. Indeed, one of the major challenges in aphasia rehabilitation is to find the persistence of gains in language and generalization to functional communication outcomes after the intervention [12].

### Conclusions

In conclusion, although several aspects need to be clarified, there are a series of advantages that make tDCS suitable to be combined with aphasia treatment. tDCS is cost-effective, very well tolerated with low adverse effects, easy-to-use, thus, it can be administered easily in a variety of settings, as during language therapy [128,165]. Moreover, the low spatial and temporal resolution which does not allow for specifically targeting a particular language area [166,167,168,169,170] might result in a further advantage. Indeed, in our opinion, the diffusion of current inside a damaged system (i.e., the left hemisphere), if it exerts its influence also far away from the targeted area, it may not be entirely negative since it might simultaneously affect several undamaged areas resulting in greatest language recovery.

## 5. General Conclusions

In this review, we have provided an overview of the most innovative approaches to post-stroke aphasia, which may overcome some limitations of traditional treatments. Indeed, as pointed out in the Introduction, there is a general agreement that treatment intensity is the most important predictor for treatment effectiveness [12]. However, for several reasons, it is not always possible to guarantee an intensive treatment for all patients [171]. A clear advantage of the above proposed approaches is that they might reinforce the effects of conventional therapies, thus, ensuring an adequate intensity of the treatment [4,89,108]

Apart from this, an interesting aspect of those techniques is that they all overcome the traditional view of language representation considering the language network much larger distributed throughout the brain than previously suggested by the classical approach [37,110,119,124,140,141,146]. Within this view, the language system closely interacts with other systems (e.g., the motor regions), thus, different part of the brain, if stimulated, might take part in language recovery. Indeed, the pharmacological approach aims to rebalance the neurotransmitter activity starting from the assumption that the same neurotransmitter might subserve different functions. Therefore, the action of the drugs will not be circumscribed into specific regions. It is likely that aphasic deficits, and probably other cognitive post-stroke deficits, partly result from damage to specific neurotransmission systems [36]. For example, it has already been suggested that damage to the cholinergic system may interfere with the processing of incoming verbal stimuli and favor the emergence of perseverations, omissions, and semantic errors in aphasia [74,172].

As previously pointed out, also the use of VR starts from the hypothesis that the language system relies on neural networks which do not necessarily comprise the classical language areas (i.e., Broca vs. Wernicke’s area). Indeed, it has been suggested that, during VR Therapy, specific motor circuits are recruited which process the sensorimotor properties of words [119,125,126]. Accordingly, tDCS has been recently applied over the motor regions with positive outcomes in language recovery [136,140,146].

Thus, we believe that these innovative approaches might result really promising for the future since they give the possibility to potentiate language recovery through stimulation of areas other than the classical ones. Indeed, given the wide variability of cortical lesions among aphasic patients, it is not always easy to localize through non-invasive brain stimulation techniques, such as transcranial direct current stimulation (tDCS), the optimal stimulation cortical sites. This points to the urgency of considering other vicarious systems, functionally connected to the brain, that, when stimulated, contribute to the recovery of language.

Unfortunately, due to the lack of a sufficient number of studies for each reported approach, we cannot reach a definitive conclusion on its real effectiveness. We urgently need to promote large clinical randomized trials in order to understand which is the best approach and, more importantly, which patients are likely to benefit.

For future research, an interesting possibility could be to adopt a mixed approach. Indeed, for motor recovery, different studies have already reported positive effects on the combined use of VR and tDCS [173,174,175,176] and VR with tele-practice [177]. In addition, as previously described (Section 2.2), since the combined effect of d-amphetamine with tDCS and language therapy was already successfully investigated in post-stroke aphasia, it would also be interesting to further explore this new approach [61].

## Figures and Tables

**Table 1:** Pharmacological studies in aphasia recovery. Abbreviations: * RCT: randomized controlled trial; SC: single case; CS: case series; OLT: Open-label trial; CT: Controlled- Trial not randomized; ERP: event-related.

Articles *	Type of Drug and Dose	Rationale/Effect	Study Design	Patients	Task	Trial Duration	Side Effects	Effects
Güngör et al., 2011 [38]	Piracetam (4800 mg/day)	It reestablishes cell membrane fluidity. It enhances neuroplasticity and neuroprotective action	RCT	30 acute *	No	6 months	Gastrointestinal irritability, nausea, vomiting, anxiety, irritability, agitation, seizures	Improvement in auditory comprehension
Kessler et al., 2000 [39]	Piracetam (4800 mg/day)	-	RCT	24 acute	Intensive language therapy; occupational therapy	6 weeks	Not reported	Improvement in spontaneous speech
Huber et al., 1997 [40]	Piracetam (4800 mg/day)	-	RCT	50 acute	Intensive language therapy (symptom-specific training)	6 weeks	No	Improvement in written language
Enderby et al., 1994 [41]	Piracetam (4800 mg/day)	-	RCT	137 acute	Speech therapy (unspecified tasks)	12 weeks	Sleep disturbances, vertigo, tiredness	Improvement in repetition and written language
Dávila et al., 2020 [42]	Donepezil (up to 5 mg/day)	It increases the cholinergic transmission. It enhances attention and learning.	SC	1 chronic	Drug alone; Intensive Naming Therapy (INT)	32 weeks	No	Drug alone: improvement in fluency and spontaneous speech. Drug + INT: improvement in naming
Berthier et al., 2017 [43]	Donepezil (up to 10 mg/day)	-	SC	1 chronic	Drug alone; Audiovisual repetition-imitation therapy	4 months	Not reported	Improvement in speech production and phrase repetition (both drug alone and drug + audiovisual repetition-imitation therapy)
Woodhead et al., 2017 [44]	Donepezil (up to 10 mg/day)	-	RCT	20 chronic	Phonological training (“Farobics”) software	25 weeks	Insomnia, headaches, dizziness, muscle cramps	Worsening in comprehension
Yoon et al., 2015 [45]	Donepezil (up to 10 mg/day)	-	SC	1 sub-acute/chronic (two infarction: 8-years before and 4 months before)	No	12 weeks	Not reported	Improvement in comprehension and spontaneous speech
Berthier et al., 2006 [46]	Donepezil (up to 10 mg/day)	-	RCT	26 chronic	Speech-Language Therapy (SLT) (unspecified tasks)	20 weeks	Irritability, insomnia, tiredness	Improvement in picture naming
Berthier et al., 2003 [47]	Donepezil (up to 10 mg/day)	-	OLT	11 chronic	Speech-Language Therapy (SLT) (unspecified tasks)	20 weeks	Irritability, increased sexual drive	Improvement in phonemic discrimination of no words, repetition, word-picture matching
Hong et al., 2012 [48]	Galantamine (up to 16 mg/day)	It increases glutamate activity. It enhances cognition, learning and memory.	OLT	45 chronic	No	12 weeks	Not reported	Improvement in spontaneous speech, comprehension, and naming
Barbancho et al., 2015 [49]	Memantine (10 mg/day)	It reduces glutamate activity (neuroprotection).	RCT	28 chronic	Drug alone; Constraint-Induced Aphasia Therapy (CIAT)	20 weeks	No	General improvement in the severity of aphasia (in both conditions)
Berthier et al., 2009 [50]	Memantine (20 mg/day)	-		28 chronic	Constraint-Induced Aphasia Therapy (CIAT)	48 weeks	No	Improvement in spontaneous speech, comprehension, naming, and everyday communication
Breitenstein et al., 2015 [51]	L-dopa (100 mg/day)	Dopamine precursor, it modulates attention, working memory, LTP, neuronal growth.	RCT	20 chronic	Conversational training; naming exercises;	6 months	Not reported	No significant effect
Leemann et al., 2011 [52]	L-dopa (100 mg/day)	-	RCT	17 sub-acute	Computer-Assisted Therapy (CAT)	2 weeks	Not reported	No significant effect
Seniów et al., 2009 [53]	L-dopa (100 mg/day)	-	RCT	39 acute/sub-acute (2 to 8 weeks post-onset)	Speech-Language Therapy (SLT) (unspecified tasks)	3 weeks	No	Improvement in verbal fluency and repetition
Ashtary et al., 2006 [54]	Bromocriptine (up to 10 mg/day)	It stimulates D2 dopamine receptor non adenyl cyclase-linked. It enhances dopamine transmission	RCT	38 acute	No	4 months	No	No significant effect
Raymer et al., 2001 [55]	Bromocriptine (up to 20 mg/day)	-	SC	1 sub-acute	No	2 months	Not reported	Improvement in verbal fluency
Gold et al., 2000 [56]	Bromocriptine (up to 15 mg/day)	-	OLT	4 sub-acute	No	8 weeks	Not reported	Improvement in word retrieval
Bragoni et al., 2000 [57]	Bromocriptine (up to 30 mg/day) + domperidon	-	CT	11 chronic	Drug alone; Speech Therapy (ST) (unspecified tasks)	4 months	Epileptic seizures, atrial flutter, atrial fibrillation, visual hallucinations, mild nausea, syncopal episode	Drugs alone: improvement in reading comprehension and verbal latency. Drugs + speech therapy: improvement in reading comprehension, repetition, and verbal latency
Sabe et al., 1995 [58]	Bromocriptine (up to 60 mg/day)	-	RCT	7 chronic	No	6 weeks	Dystonic movements (hemiparetic side), nausea, lack of energy	No significant effect
Gupta et al. 1995 [59]	Bromocriptine (up to 15 mg/day)	-	CT	28 chronic	No	28 weeks	Nausea, painful dystonia	No significant effect
Gupta and Mlcoch, 1992 [60]	Bromocriptine (up to 30 mg/day)	-	OLT	2 chronic	No	3 months	Not reported	Improvement in mean length utterance, verbal fluency, and naming
Keser et al., 2017 [61]	d-Amphetamine (10 mg, two doses)	It increases dopamine activity. It enhances vigilance and attention. It is involved in synaptogenesis.	RCT	10 chronic	Melodic Intonation Therapy (MIT)	2 days (separated by 10 washout days)	Mild insomnia	General improvement in aphasia severity
Spiegel and Alexander, 2011 [62]	Amphetamine and d-Amphetamine (up to 10 mg/day)	-	SC	1 (stroke stage not reported)	Speech therapy (unspecified tasks)	3 weeks	No	Improvement in naming and repetition
Whiting et al., 2007 [63]	d-Amphetamine (5 mg/day)	-	CT	2 chronic	Computer-based therapy	1 month	No	No significant effect
Stefanatos et al., 2006 [64]	d-Amphetamine (20 mg, single dose)	-	CT	10 sub-acute	No	1 day	Not reported	Improvement in auditory attention
Walker-Batson et al., 2001 [65]	d-Amphetamine (10 mg/day)	-	RCT	21 acute	Speech-Language Therapy (SLT) (individualized, based on traditional stimulation/facilitation model)	6 months	No	General improvement in aphasia severity
McNeil et al., 1997 [66]	Selegiline (up to 20 mg/day)	“	CT	2 chronic	Lexical-Semantic Activation Inhibition Treatment (L-SAIT)	21 days	Not reported	No significant effect
Walker-Batson et al., 1992 [67]	d-Amphetamine (up to 15 mg/day)	-	OLT	6 acute	Speech-Language Therapy (SLT) (in auditory comprehension, speaking, reading, and writing)	3 months	Mild insomnia	No significant effect
Walker-Batson et al., 1991 [68]	d-Amphetamine (10 mg/day)	-	SC	1 acute	Speech-Language Therapy (SLT) (contrastive stress drills coupled with traditional speaking, reading, and auditory comprehension tasks)	11 months	Not reported	General improvement in aphasia severity, in particular in verbal fluency

* Acute (< four weeks post-onset); Sub-Acute (>four weeks post-onset); Chronic (>one year post-onset).

**Table 2:** Virtual Reality studies in aphasia recovery. Abbreviation: * RCT: randomized controlled trial; CS: case series; RGSa: Rehabilitation Gaming System for aphasia Interaction; VRRS-Tablet: Virtual Reality Rehabilitation System; RGS Rehabilitation Gaming System; VR: virtual reality.

Articles *	Study Design	Patients	Task	Technology	Dose/Intensity	Effects
Giachero et al., 2020 [119]	RTC	36 chronic *	Conversational Training Therapy (CTT)	VR (NeuroVR 2.0)	Two-hour therapy session, twice a week for 24 weeks.	VR improved oral comprehension, repetition, written language, self-esteem, and emotional state
Marshall et al., 2020 [120]	RTC	34 aphasics	Group Therapy	VR (EVA Park)	Twenty-one hours therapy session for six months; sessions occurring once in a fortnight.	No significant improvement in wellbeing, communicative success
Grechuta et al., 2019 [110]	RTC	17 chronic	Intensive Language-Action Therapy (ILAT)	RGSa	Five half-hour therapy session for eight weeks.	Improvement in speech production, auditory comprehension, communicative effectiveness in everyday life, and lexical access
Maresca et al., 2019 [121]	RTC	30 subacute	Naming, composition, writing and rewriting	VRRS-Tablet	Five fifty-minutes sessions for two weeks.	VRRS-Tablet Significant improved comprehension, repetition, reading, calculation, competence, adaptability, and self-esteem
Marshall et al., 2018 [122]	CS	2 chronic	Noun retrieval and Verb Network Strengthening	VR (EVA Park)	Twenty- one-hour therapy session over five weeks.	VR improved noun retrieval
Carragher et al., 2018 [123]	CS	3 chronic	Storytelling	VR (EVA Park)	Twenty one-hour therapy session over five weeks.	VR improved storytelling
Grechuta et al., 2017 [124]	Longitudinal trial	4 chronic	Sensorimotor speech therapy	RGS	Five forty- minutes therapy session over eight weeks.	RGS improved word retrieval and verb execution
Marshall et al., 2016 [113]	quasi-RTC	20 chronic	Free communication	VR (EVA Park)	Twenty-five one-hour therapy session over five weeks.	VR improved functional communication

* Acute (<four weeks post-onset); Sub-Acute (>four weeks post-onset); Chronic (>one year post-onset).

**Table 3:** Transcranial direct current stimulation studies in aphasia recovery. Abbreviation: L left; R: right; IFG: inferior frontal gyrus; ST: superior temporal; M1: motor cortex; PPR: posterior perisylvian region; DLPFC: dorsolateral-prefrontal cortex, Bihemisph: bihemispheric; Generaliz: generalization; Periles: perilesional; Individ: Individualized.

Articles	Patients	Task	tDCS vs. Sham	Target (Areas)	tDCS Stimulation Parameters	Generaliz.	Follow-Up	Effects
Guillouët et al., 2020 [132]	14 aphasics (7 non-fluent, 4 fluent 3 mixed), but 10 patients completed the study	Personalized speech and language therapy	No	Bihemisph Anode: L IFG Cathode: R IFG	2 mA, 20 min 2–5 sessions a week for 3 weeks	No	No	no improvement in spontaneous speech
Fiori et al., 2019 [133]	2 groups of 10 non-fluent aphasics	Verb naming	Yes, only after cathodal stimulation at 2 mA	Cathode: R IFG	1 mA, 20 min 2 mA, 20 min 5 sessions	Not investigated	Yes, at 1 week	improvement in verb recovery
Stahl et al., 2019 [134]	130 aphasics	Computer-assisted naming therapy and face-to-face communicative-pragmatic therapy	Yes	Anode: L M1	1 mA, 20 min Two daily sessions over three consecutive weeks	Yes	Yes, at 6 months and 12 months	improvement in communicative ability
Vila-Nova et al., 2019 [135]	12 (5 non-fluent, 5 fluent, 2 transcortical not defined)	Articulatory accuracy, syllable repetition, and word production	Yes, only for articulatory accuracy	Anode: L IFG	1 mA, 20 min 5 sessions	No	Yes, at 1 week and 4 months qualitative follow up	improvement in articulatory accuracy
Branscheidt et al., 2018 [136]	16 (6 non-fluent, 5 fluent, 5 mixed)	Lexical decision task	Yes	Anode: L M1	2 mA, 20 min 1 session	No	No	improvement of accuracy in lexical decision, especially for action words
Silva et al., 2018 [137]	14 non-fluent	Naming task	Yes, only at Boston test (mean time for correct responses with strategy)	Cathode: R IFG	2 mA, 20 min 5 sessions	No	Yes, at 4 weeks	only improvement in Boston test performance
Fridriksson et al., 2018 [138]	74 (43 non-fluent, 31 fluent)	Computerized behavioral treatment of anomia	Yes	Anode: L Temporal cortex	1 mA, 20 min 15 sessions	Yes	Yes, at 6 months	improvement in anomia
Fridriksson et al., 2018 [139]	74 aphasics	Computerized picture-word matching task	Yes	Anode: L Temporal cortex	1 mA, 20 min 15 sessions	Yes	Yes, at 4 weeks and 24 weeks	improvement in naming
Spielmann et al., 2018 [19]	13 aphasics	Word finding Therapy	Yes	Anode: L IFG or L ST	1 mA; 20 min 3 sessions	No	No	improvement in word finding
Marangolo et al., 2018 [140]	12 non-fluent	Verb naming and verb generation	Yes, only for verb generation	Cathode: R cerebellar cortex	2 Am, 20 min 5 sessions	No	Yes, at 1 week	improvement in verb generation
Pestalozzi et al., 2018 [141]	14 (10 non-fluent, 4 fluent)	Picture naming, phonemic fluency task, and repetition task	Yes	Anode: L DLPFC	1 mA, 20 min 1 session	No	No	improvement in high-frequency word fluency
Woodhead et al., 2018 [142]	21 aphasics with central alexia	iReadMore training	Yes	Anode: L IFG	2 mA, 20 min 5 sessions	Yes	Yes, at 3 months	facilitation in reading ability
Darkow et al., 2017 [143]	16 mild aphasics	Noun picture naming	No	Anode: L M1	1 mA, 20 min 1 session	Not investigated	No	No differences between real and sham condition;
Santos et al., 2017 [144]	13 non-fluent	Picture naming	No	Anode: L IFG	2 mA, 20 min 1 session	No	No	No improvement in picture naming
Norise et al., 2017 [145]	9 non-fluent	Noun picture naming based on constraint-induced language therapy (CILT)	Yes	Anode/Cathode: L IFG Anode/Cathode: RIFG	2 mA, for 20 min 10 sessions	No	Yes, at 2 weeks and 2 months	improvement in speech fluency
Marangolo et al., 2016 [26]	9 non-fluent	Repetition	Yes	Bihemisph Anode: L IFG Cathode: R IFG	2 mA, 20 min 15 sessions	Yes	Yes, at 1 week	improvement in speech articulation
Meinzer et al., 2016 [146]	26 (15 non-fluent, 11 fluent)	Computer-assisted naming treatment	Yes	Anode: L M1	1 mA, 20 min for Two daily treatment for 8 sessions	Yes	Yes, at 6 months	improvement in naming
Campana et al., 2015 [27]	20 non-fluent	Conversational therapy	Yes	Anode: L IFG	2 mA, 20 min 10 sessions	Not investigated	No	improvement in picture description, noun and verb naming
Cipollari et al., 2015 [147]	6 non-fluent	MIT	Yes	Anode: R IFG	2 mA, 20 min 15 sessions	Yes	Yes, at 1 week	improvement in repetition accuracy for words and sentences.
de Aguiar et al., 2015 [148]	9 (3 fluent, 6 non fluent)	Verb treatment “ACTION”	Yes	Bihemisph. Anode and Cathode: left periles. area	1 mA, 20 min 10 sessions	Yes	No	improvement in verb production
Richardson et al., 2015 [149]	8 non-fluent	Computerized noun naming	Yes	Individ. electrode placement by fMRI	1 mA, 20 min 22 sessions spread out in 5 weeks	No	Yes, at 1 week	Improvement in naming accuracy after HD-tDCS and CS-tDCS
Shah-Basak et al., 2015 [20]	12 non-fluent	Picture naming	Yes	Anode: L/R DLPFC, Cathode: L/R DLPFC	2 mA, 20 min 10 sessions	Yes	Yes, at 2 weeks and 2 months	improvement in naming
Marangolo et al., 2014a [29]	7 non-fluent	Conversational therapy	Yes	Bihemisph Anode: L IFG Cathode: R IFG	2 mA, 20 min 10 sessions	Yes	Yes, at 1 week	improvement in picture description, noun and verb naming
Marangolo et al., 2014b [30]	8 non-fluent	Conversational therapy	Yes	Anode: L IFG or L ST	1 mA, 20 min 10 sessions	Yes	No	improvement in cohesive elements (pronouns, ellipses, word repetitions, conjunctions) after a-tDCS over LIFG.
Vestito et al., 2014 [150]	3 (2 non-fluent aphasics, 1 fluent anomic)	Noun and Verb picture naming	Yes	Anode: L IFG	1.5 mA, 20 min 10 sessions	Yes	Yes, at 6 weeks	improvement in naming
Fiori et al., 2013 [21]	7 non-fluent	Noun and Verb picture Naming	Yes	Anode: L IFG or L ST	1 mA, 20 min 5 sessions	Yes	Yes, at 1 week and 4 weeks	A-tDCS over L IFG improved verb naming; A tDCS over L ST improved noun naming
Lee et al., 2013 [151]	11 (6 non-fluent and 5 fluent)	Picture naming, reading short paragraphs	Yes	Anode: L IFG or Bihemisph Anode: L IFG Cathode: R IFG	2 mA, 30 min 1 session	Not investigated	No	Improvement in naming accuracy after bihemispheric and single tDCS.
Marangolo et al., 2013 a [25]	12 non-fluent	Repetition	Yes	Bihemisph Anode: L IFGCathode: R IFG	1 mA, 20 min 5 sessions	Yes	Yes at 1 week	improvement in speech articulation
Marangolo et al., 2013 b [28]	12 non-fluent	Conversational therapy	Yes	Anode: L IFG, L ST	1 mA, 20 min 10 sessions	Yes	Yes, at 4 weeks	improvement in informative speech, content units, verbs, and sentences
Marangolo et al., 2013 c [23]	8 non-fluent	Picture naming	Yes	Anodal: L IFG or L ST	1 mA, 20 min 5 sessions	Not reported	Yes, at 4 weeks	improvement in verb naming after A-tDCS over the LIFG
Flöel et al., 2011 [152]	12 (9 non-fluent and 3 fluent)	Computerized picture naming	Yes	Anode vs. Cathode over R TP cortex;	1 mA, 20 min 3 session	No	Yes, at 2 weeks	improvement in picture naming
Fridriksson et al., 2011 [153]	8 fluent	Computerized picture naming	Yes	Anode: L posterior cortex	1 mA, 20 min 5 sessions	No	Yes, at 3 weeks	improvement in picture naming
Fiori et al., 2011 [22]	3 non-fluent	Noun picture naming	Yes	Anode: L ST	1 mA, 20 min 5 sessions	Not reported	Yes, at 3 weeks	improvement in picture naming
Kang et al., 2011 [154]	10 (8 non-fluent and 2 fluent)	Noun picture naming	Yes	Cathode: R IFG	2 mA, 20 min 5 sessions	No	No	improvement in picture naming
Marangolo et al., 2011 [24]	3 non fluent	Repetition	Yes	Anode: L IFG	1 mA, 20 min 5 sessions	Yes	Yes, at 1 week and at 2 months	improvement in speech accuracy and fluency
Vines et al., 2011 [155]	6 non-fluent	MIT	Yes	Anode: R IFG	1.2 mA, 20 min 3 sessions	Yes	No	improvement in speech fluency
Baker et al., 2010 [156]	10 (6 fluent and 4 non-fluent)	Computerized picture naming	Yes	Anode: L F	1 mA, 20 min 5 sessions	Yes	Yes, at 1 week	improvement in picture naming
